# Reversal of ABCB1-related multidrug resistance by ERK5-IN-1

**DOI:** 10.1186/s13046-020-1537-9

**Published:** 2020-03-12

**Authors:** Fang Wang, Delan Li, ZongHeng Zheng, Kenneth Kin Wah To, Zhen Chen, Mengjun Zhong, Xiaodong Su, Likun Chen, Liwu Fu

**Affiliations:** 1 State Key Laboratory of Oncology in South China, Collaborative Innovation Center for Cancer Medicine, Sun Yat-sen University Cancer Center, Guangzhou, 510060 People’s Republic of China; 2 Department of Gastrointestinal surgery, The Third Affiliated Hospital of Sun Yat-sen University, Guangzhou, 510000 Guangdong People’s Republic of China; 3 School of Pharmacy, the Chinese University of Hong Kong, Hong Kong, People’s Republic of China

**Keywords:** ERK5-IN-1, Multi-drug resistance, ATP-binding cassette transporter, ABCB1

## Abstract

**Background:**

Inhibition of ABC transporters is considered the most effective way to circumvent multidrug resistance (MDR). In the present study, we evaluated the MDR modulatory potential of ERK5-IN-1, a potent extracelluar signal regulated kinase 5 (ERK5) inhibitor.

**Methods:**

The cytotoxicity and MDR reversal effect of ERK5-IN-1 were assessed by MTT assay. The KBv200-inoculated nude mice xenograft model was used for the in vivo study. Doxorubicin efflux and accumulation were measured by flow cytometry. The modulation of ABCB1 activity was measured by colorimetric ATPase assay and [^125^I]-iodoarylazidoprazosin (IAAP) photolabeling assay. Effect of ERK5-IN-1 on expression of ABCB1 and its downstream markers was measured by PCR and/or Western blot. Cell surface expression and subcellular localization of ABCB1 were tested by flow cytometry and immunofluorescence.

**Results:**

Our results showed that ERK5-IN-1 significantly increased the sensitivity of vincristine, paclitaxel and doxorubicin in KBv200, MCF7/adr and HEK293/ABCB1 cells, respectively. This effect was not found in respective drug sensitive parental cell lines. Moreover, in vivo combination studies showed that ERK5-IN-1 effectively enhanced the antitumor activity of paclitaxel in KBv200 xenografts without causing addition toxicity. Mechanistically, ERK5-IN-1 increased intracellular accumulation of doxorubicin dose dependently by directly inhibiting the efflux function of ABCB1. ERK5-IN-1 stimulated the ABCB1 ATPase activity and inhibited the incorporation of [^125^I]-iodoarylazidoprazosin (IAAP) into ABCB1 in a concentration-dependent manner. In addition, ERK5-IN-1 treatment neither altered the expression level of ABCB1 nor blocked the phosphorylation of downstream Akt or Erk1/2. No significant reversal effect was observed on ABCG2-, ABCC1-, MRP7- and LRP-mediated drug resistance.

**Conclusions:**

Collectively, these results indicated that ERK5-IN-1 efficiently reversed ABCB1-mediated MDR by competitively inhibiting the ABCB1 drug efflux function. The use of ERK5-IN-1 to restore sensitivity to chemotherapy or to prevent resistance could be a potential treatment strategy for cancer patients.

## Background

Despite considerable advances in chemotherapies against cancer, multi-drug resistance (MDR) remains a major obstacle to positive therapeutic outcomes [1, 2]. One of the major causes of MDR involves the increased expression of drug efflux transporters of the ATP binding cassette (ABC) family [3–6]. Structurally, ABC transporters are highly conserved and each is composed of at least one hydrophobic membrane-spanning domain (MSD). By coupling ATP binding and hydrolysis, these pumps reduce the intracellular accumulation of many anticancer drugs to sub-therapeutic levels, thus decreasing or abolishing chemotherapy efficacy [7, 8]. To date, at least 15 ABC transporters have been implicated to confer resistance to cancer chemotherapy, notably P-glycoprotein (P-gp, ABCB1) and breast cancer resistance protein (BCRP, ABCG2), are overexpressed in various cancers and extrude a wide range of chemotherapeutic agents, making them attractive therapeutic targets [9–15].

The most direct way to restore drug sensitivity in MDR cancer cells caused by ABC transporters is to block or modulate their activity [16]. So far, 4 generations of ABC transporter modulators including verapamil, valspodar, tariquidar, Fumitremorgin C and Ko143 have been developed [17, 18]. Unfortunately, Clinical trials using MDR-inhibitors have had only limited success, mainly due to significant toxicity, lack of specificity and drug-interactions [19–21]. These limitations have spurred efforts to search for safe and effective inhibitors of these transporters.

Small molecule tyrosine kinase inhibitors (TKIs) are developed to block the uncontrolled activity of intracellular signaling pathways in tumor cells. Our studies have previously revealed that some TKIs are either inhibitors or substrates to alter efflux mechanisms mediated by ABC transporters. TKIs such as EGFR inhibitors (erlotinib, lapatinib), VEGFR inhibitors (axitinib, apatinib) and others can significantly inhibit the efflux function of ABC transporters, thereby sensitizing cancer cells to chemotherapeutic agents [22–26].

ERK5-IN-1, benzo [e] pyrimido-[5, 4-b] diazepine-6 (11H)-one was discovered as a novel extracelluar-signal-regulated kinase 5 (ERK5) inhibitor [27]. ERK5, also known as big mitogen-activated protein kinase-1 (BMK1), has been proposed as an interesting target to tackle different cancers [28–30]. Either kinase inhibitors or down-regulating protein expression displayed antiproliferative activity against cancer cells [31]. In the present study, we evaluated the MDR reversing potential of ERK5-IN-1, to discover new indications for this compound in preventing chemotherapeutic drug resistance in cancer patients.

## Methods

### Chemicals and reagents

ERK5-IN-1 was purchased from Selleck Chemicals (Houston, TX, USA). 1-(4, 5-dimethylthiazol-2-yl)-3, 5-diphenylformazan (MTT), vincristine, paclitaxel, verapamil, cisplatin, doxorubicin (Dox), mitoxantrone, fumitremorgin C (FTC) and other chemicals were purchased from Sigma-Aldrich (St. Louis, MO, USA). DMEM and RPMI 1640 medium were obtained from Thermo Fisher Scientific Inc. (Waltham, MA, USA). Antibodies against ABCB1/P-gp (sc-13,131), Akt (sc-8312), p-Akt (sc-7985-R), ERK 1/2 (sc-514,302), and p-ERK (sc-7383) were from Santa Cruz Biotechnology Inc. (Paso Robles, CA, USA). ABCG2 antibody (MAB4146) was obtained from Chemicon International, Inc. (Billerica, MA). Glyceraldehyde-3-phosphate dehydrogenase (GAPDH) antibody was from Kangcheng Co. (Shanghai, China). SYBR Green qPCR Master Mix was from ExCell Bio (Shanghai, China). Flow cytometry antibodies against ABCB1 (#557002) and Mouse IgG2b/κ (#559532) were purchased from BD Biosciences (San Jose, CA, USA).

### Cell lines and cell culture

The human oral epidermoid carcinoma cell KB and its VCR-selected ABCB1-overexpressing KBv200 cells; human leukemia cell HL60 and its doxorubicin-selected ABCC1 overexpressing HL60/adr cells were maintained in complete medium consisting of RPMI 1640 supplemented with 10% fetal bovine serum (FBS), 100 U/mL penicillin and 100 μg/mL streptomycin at 37 °C in the presence of 5% CO_2_ [32]. Human colon carcinoma cell S1 and its MX-selected ABCG2-overexpressing S1-MI-80 cells [33]; human breast carcinoma cell MCF-7 and its Dox-selected ABCB1-overexpressing MCF-7/adr cells [34]; human lung squamous carcinoma cell SW1573 and its doxorubicin-selected LRP overexpressing SW1573/2R120 cell; HEK293 and its ABCB1 and MRP7 stable gene-transfected HEK293/ABCB1 and HEK293/MRP7–2 cells were cultured in DMEM supplemented with 10% fetal bovine serum (FBS), 100 U/mL penicillin and 100 μg/mL streptomycin at 37 °C in the presence of 5% CO_2_ [35].

### Cell cytotoxicity assay

MTT assay was used to assess the cytotoxicity of ERK5-IN-1 and chemotherapeutic agents, individually or in combination as described previously [36]. Bliss method was used to calculate the half maximal (50%) inhibitory concentration (IC_50_) values. The fold-reversal of MDR was calculated by dividing the IC_50_ of chemotherapeutic agents in the absence of ERK5-IN-1 by that obtained in the presence of ERK5-IN-1.

### Animal experiments

The KBv200-inoculated nude mice xenograft model was used for the in vivo study. Briefly, 2.5 × 10^6^ KBv200 cells were subcutaneously injected into the right flank of mice (Athymic nude mice, 4 to 6 weeks old, weighing 16 to 18 g). When the tumors reached a mean volume of 50mm^3^, mice were randomized into four groups and received various treatments: (a) saline (q3d); (b) paclitaxel (20 mg/kg, i.p. q3d); (c) ERK5-IN-1 (25 mg/kg, p.o. q3d); (d) ERK5-IN-1 (25 mg/kg, p.o. q3d, given 1 h before paclitaxel administration) plus paclitaxel (20 mg/kg, i.p. q3d). The body weight and two perpendicular tumor diameters (A and B) were measured every 2 days, and the tumor volume (V) was calculated according to the following formula: V = (π/6)[(A + B)/2]^3^. When the average tumor volume reached 2000 mm^3^ in the saline group, mice were euthanized and the xenografts were excised and weighed. The ratio of tumor growth inhibition (IR) was estimated according to the following formula [37].

$$ IR\left(\%\right)=\left(1-\frac{\mathrm{Mean}\ \mathrm{tumor}\ \mathrm{weight}\ \mathrm{of}\ \mathrm{control}\ \mathrm{group}}{\mathrm{Mean}\ \mathrm{tumor}\ \mathrm{weight}\ \mathrm{of}\ \mathrm{experiment}\ \mathrm{group}}\right)\times 100 $$

All experiments were conducted with the approval of the Sun Yat-sen University.

Institutional Animal Care and Use Committee.

### Dox accumulation assay

The effect of ERK5-IN-1 on the intracellular accumulation of Dox was measured by flow cytometry as previously described [24]. Briefly, cells were incubated with different concentrations of ERK5-IN-1 or vehicle at 37 °C for 3 h. Then, 10 μM Dox was added and cells were cultured for another 3 h. Finally, the cells were collected, washed 3 times with chilled phosphate-buffered saline (PBS) and analyzed by flow cytometry (Cytomics FC500, Beckman Coulter Inc., Brea, CA, USA). VRP and FTC were used as positive control.

### Dox efflux assay

Dox efflux assay was performed as described formerly [37]. KB and KBv200 cells were treated with 10 μM Dox at 37 °C for 3 h. Then the cells were washed three times with PBS and incubated at 37 °C with fresh media in the presence or absence of 0.4 μM ERK5-IN-1. Cells were collected at indicated time points (0, 15, 30, 60, 90 and 120 min), resuspended in chilled PBS and analyzed by flow cytometry (Cytomics FC500, Beckman Coulter Inc., Brea, CA, USA).

### ABCB1 ATPase assay

A colorimetric ATPase assay was performed as previously described with minor modification [38]. Briefly, crude membranes isolated from High Five insect cells expressing ABCB1 (100 μg protein/mL) were incubated at 37 °C with different concentrations of ERK5-IN-1 in the presence or absence of sodium orthovanadate (0.3 mM) in ATPase assay buffer (50 mM KCl, 5 mM sodium azide, 2 mM EDTA, 10 mM MgCl_2_, 1 mM DTT, pH 6.8) for 5 min. ATP hydrolysis reaction was then started by the addition of 5 mM Mg-ATP and incubated for 20 min. Luminescence was initiated by detection buffer (35 mM ammonium molybdate, 15 mM zinc acetate, 10% ascorbic acid). After incubation at 37 °C for another 20 min, absorbance was subsequently measured at 750 nm. Specific ERK5-IN-1-stimulated ABCB1 ATPase activity (i.e. vanadate-sensitive) was determined as the difference between the amounts of inorganic phosphate released from ATP in the absence and presence of sodium orthovanadate.

### Photo-affinity labeling of ABCB1 with [^125^I]-iodoarylazidoprazosin (IAAP)

Crude membrane from High Five insect cells expressing ABCB1 (50 μg) was incubated with 0–5 μM ERK5-IN-1 for 5 min at room temperature in 50 mM Tris-HCl (pH 7.5). [^125^I]-IAAP (2200 Ci/nmole, 3 nM) was then added and incubated for another 5 min under subdued light. The samples were cross-linked by UV illumination (365 nm) on ice and labeled ABCB1 was immunoprecipitated using the C219 antibody. Finally, samples were subjected to SDS-PAGE using a 7% Tris-acetate NuPAGE gel, dried and exposed to Bio-Max MR film (Eastman Kodak Co., Rochester, NY) at -80 °C for 4 h. The radioactivity incorporated into the transporter protein was quantified using the Storm 860 PhosphorImager system (Molecular Dynamics, Sunnyvale, CA).

### Western blot analysis

Western blot was performed as previously described [39]. To determine whether ERK5-IN-1 affects the expression of ABCB1, cells were pretreated with different concentrations of ERK5-IN-1 for 48 h. To evaluate whether ERK5-IN-1 blocks Akt or Erk1/2 phosphorylation at reversal concentrations, cells were incubated with ERK5-IN-1 for 24 h. Equal amounts of proteins were separated by SDS-PAGE gel and GAPDH was used as loading control.

### Reverse transcription-polymerase chain reaction (PCR) and real-time quantitative PCR

Cells were pre-treated with indicated concentrations of ERK5-IN-1 for 48 h and total cellular RNA was isolated by Trizol RNA extraction reagent (#15596026, Thermo Fisher Scientific Inc., MA, USA). The first strand cDNA was synthesized by cDNA reverse transcription Kit (Promega Corp., WI, USA) under the guidance of instructions. The PCR primers used were 5^′^-CAGGCTTGCTGTAATTACCCA-3^′^ (forward) and 5^′^-TCAAAGAAACAACGGTTCGG-3^′^ (reverse) for ABCB1; 5^′^-GAGTCAAGGATTTGGTCGT-3^′^ (forward) and 5′-GATCTCGCTCCTGGAAGATG-3′ (reverse) for GAPDH, respectively. Amplification reactions were carried out at 94 °C for 3 min for initial denaturation, and then at 94 °C for 30 s, 58 °C for 30 s and 72 °C for 1 min. After 35 cycles of amplification, additional extensions were carried out at 72 °C for 10 min. The products were resolved and examined by 1.5% agarose gel electrophoresis.

Real-time qPCR was performed using SYBR Green qPCR Master Mix according to manufacturer’s protocol. The reaction was under the following conditions: 50 °C for 2 min, 95 °C for 5 min and 40 cycles at 95 °C for 15 s, 60 °C for 30 s. Data was analyzed using the 2^-ΔΔCt^ method after normalization with the GAPDH expression level in each sample.

### Cell surface expression of ABCB1 examined by flow cytometry

Cells were treated for 48 h with ERK5-IN-1 at 0.4 μM and then collected and washed three times with chilled PBS (supplemented with 0.5% bovine serum albumin). 1 × 10^6^ cells (25 μL) were incubated with 10 μL FITC-conjugated ABCB1 antibody for 45 min at 4 °C, washed twice and resuspended in 400 μL PBS. Isotype control samples were treated with mouse IgG2b/κ antibody in parallel. All stained samples were analyzed by flow cytometry.

### Immunofluorescence and confocal microscopy

KBv200 cells seeded at 5 × 10^5^ per well on glass bottom cell culture dish and treated for 48 h in the absence and presence of ERK5-IN-1 at 0.4 μM. Cells were then washed three times with cold PBS and fixed with 4% paraformaldehyde for 15 min at room temperature and permeabilized with 0.1% Triton X-100. Subsequently, cells were washed three times with PBS, blocked for 1 h with 1% BSA in PBST and immunolabeled with ABCB1 antibody at 1:100 (cell signaling, #13342) overnight. Finally, cells were incubated with second antibody Alexa Fluor Plus 594-conjugated goat anti-rabbit IgG at 1:500 (invitrogen, A32740) for 1 h. Nuclei were counterstained with the blue fluorescent DNA stain 4′, 6-diamidino-2-phenylindole (DAPI, 1:1000; Invitrogen, Molecular Probes) for 10 min. Images were acquired by a Zeiss LSM 880 microscope using 40x oil immersion objective lens and analyzed by Zens2 Software.

### Data analysis

All experiments were repeated at least three times. The SPSS statistical software (SPSS 16.0) was used in statistical analysis. Results were shown as mean values ± standard deviation (SD) and the statistical differences were determined by using the Student’s *t*-test. The statistical significance was determined at *p* < 0.05.

## Results

### Cytotoxicity and collateral sensitivity of ERK5-IN-1

**Fig. 1:**
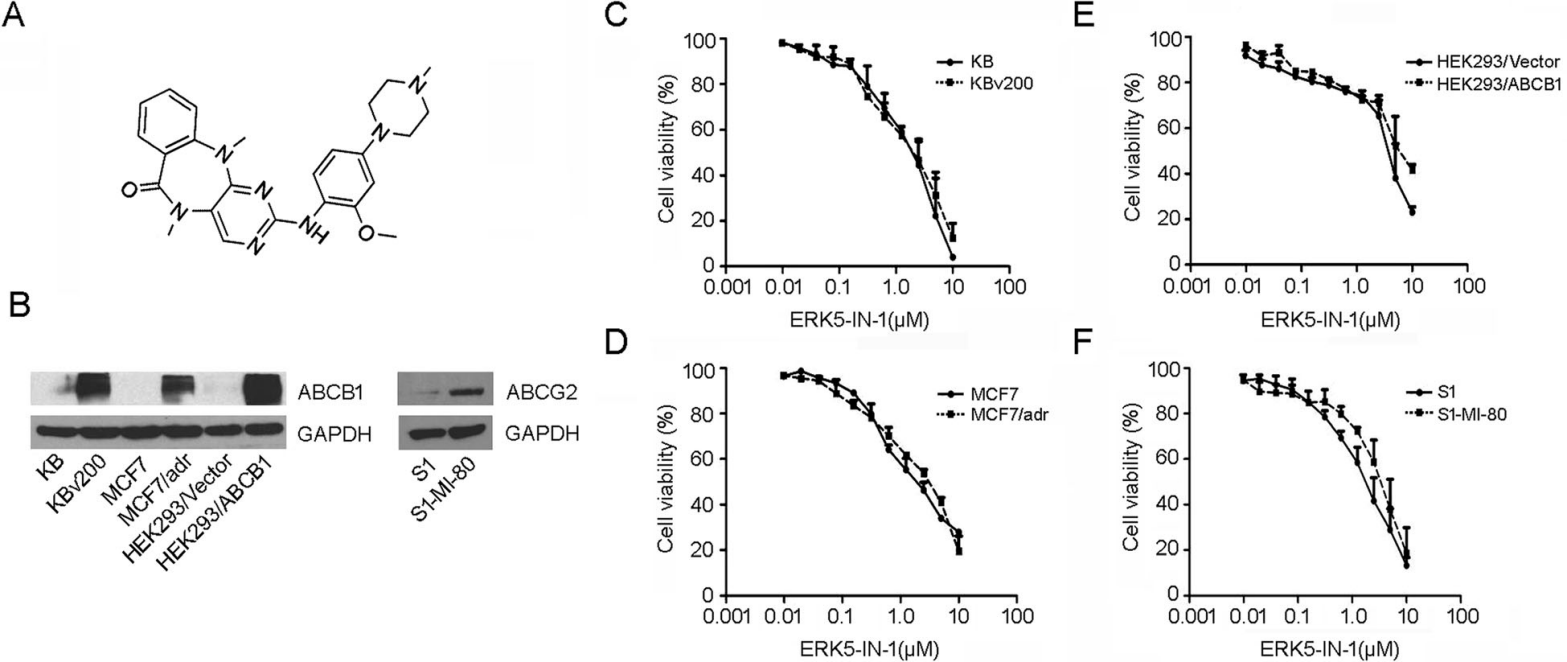
The structure and cytotoxicity effects of ERK5-IN-1. **a**, the structure of ERK5-IN-1; **b**, the protein expression of ABCB1 and ABCG2 in pairs of parental and transporter overexpressing cell lines; **c-f**, MTT cytotoxicity assay was used to measure cell survival in: **c**, KB and ABCB1-overexpressing KBv200 cells; **d**, MCF-7 and ABCB1-overexpressing MCF-7/adr cells; **e**, HEK293/Vector and HEK293/ABCB1 cells; **f**, S1 and ABCG2-overexpressing S1-MI-80 cells. All cells were exposed to the full-range concentrations of ERK5-IN-1 for 68 h. Data points represent the means ± SD of at least three independent experiments performed in triplicate

The structure of ERK5-IN-1 was shown in Fig. 1a. Western blot analysis confirmed overexpression of ABCB1 in KBv200, MCF7/adr and HEK293/ABCB1 cells. The basal expression of ABCB1 in parental KB, MCF7 and HEK293/Vector cells were nearly undetectable. ABCG2 was highly expressed in resistant S1-MI-80 cells but not in S1 cells (Fig. 1b). ERK5-IN-1 showed moderate antiproliferative and cytotoxic effects on the parental and drug-resistant cell lines. The IC_50_ values were 1.355 ± 0.077, 1.306 ± 0.052, 3.693 ± 0.097, 5.485 ± 0.366, 1.900 ± 0.175, 2.012 ± 0.648, and 1.558 ± 0.942, 2.026 ± 0.778 μM for KB, KBv200, HEK293/Vector, HEK293/ABCB1, MCF7, MCF7/adr, S1 and S1-MI-80 cells, respectively (Fig. 1c-f). Based on the cytotoxicity curves, more than 80% of cells survived when treated with ERK5-IN-1 alone up to 0.4 μM. Therefore, ERK5-IN-1 at concentrations of 0.1, 0.2 and 0.4 μM was used for combination treatment to test the reversal activity.

### ERK5-IN-1 particularly reversed ABCB1-mediated MDR in vitro

Table 1 compared the calculated IC_50_ values of chemotherapeutic agents in the presence or absence of ERK5-IN-1. Consistent with previous reports, MDR cells showed much higher resistance than the parental cells to ABCB1- or ABCG2-substrates. ERK5-IN-1 strongly enhanced the cytotoxicity of vincristine, paclitaxel and doxorubicin in KBv200, MCF7/adr and HEK293/ABCB1 cells in a dose dependent manner. No sensitization was observed in parental cells. However, a slight synergistic effect was observed in KB and HEK293/Vector cells when cells were treated with ERK5-IN-1 at high dose in combination with vincristine or doxorubicin. ERK5-IN-1 did not alter the cytotoxicity of cisplatin (non-ABCB1 substrate) in neither MDR nor drug-sensitive parental cells. Moreover, ERK5-IN-1 showed no significant effect on ABCG2-, ABCC1-, MRP7- and LRP-mediated drug resistance in S1-MI-80, HL60/adr, HEK293/MRP7–2 and SW1573/2R120 cells (Table 1 and S1).

**Table 1:** Effect of ERK5-IN-1 on enhancing efficacy of chemothereapeutic agents in ABCB1- and ABCG2-overexpressing cells

Compounds	IC_50_ ± SD (μmol/L) (fold-reversal)			
	KB		KBv200 (ABCB1)	
Vincristine	0.00131 ± 0.00009	(1.00)	0.18606 ± 0.03569	(1.00)
+ 0.1 μM ERK5-IN-1	0.00122 ± 0.00008	(1.08)	0.11322 ± 0.01575 *	(1.64)
+ 0.2 μM ERK5-IN-1	0.00074 ± 0.00010 **	(1.78)	0.07330 ± 0.00529 **	(2.54)
+ 0.4 μM ERK5-IN-1	0.00066 ± 0.00004 **	(2.00)	0.02260 ± 0.01838 **	(8.23)
+ 10 μM Verapamil	0.00141 ± 0.00025	(0.93)	0.00055 ± 0.00008 **	(337.99)
Paclitaxel	0.00329 ± 0.00055	(1.00)	0.19882 ± 0.01985	(1.00)
+ 0.1 μM ERK5-IN-1	0.00299 ± 0.00071	(1.10)	0.09600 ± 0.01963 *	(2.07)
+ 0.2 μM ERK5-IN-1	0.00346 ± 0.00075	(0.95)	0.06959 ± 0.00393 *	(2.86)
+ 0.4 μM ERK5-IN-1	0.00275 ± 0.00023	(1.20)	0.01793 ± 0.00351 **	(11.09)
+ 10 μM Verapamil	0.00306 ± 0.00021	(1.08)	0.00124 ± 0.00135 **	(160.50)
Cisplatin	0.15037 ± 0.02336	(1.00)	1.30926 ± 0.06360	(1.00)
+ 0.4 μM ERK5-IN-1	0.15123 ± 0.03312	(0.99)	1.30950 ± 0.09836	(1.00)
	MCF-7		MCF-7/adr (ABCB1)	
Doxorubicin	0.20569 ± 0.08712	(1.00)	7.17759 ± 1.37837	(1.00)
+ 0.1 μM ERK5-IN-1	0.18715 ± 0.03454	(1.10)	1.77472 ± 0.19202 **	(4.04)
+ 0.2 μM ERK5-IN-1	0.12307 ± 0.01691	(1.67)	1.32842 ± 0.77573 **	(5.40)
+ 0.4 μM ERK5-IN-1	0.12114 ± 0.04427	(1.70)	0.94483 ± 0.11191 **	(7.60)
+ 10 μM Verapamil	0.19548 ± 0.01487	(1.05)	0.27687 ± 0.05879 **	(25.92)
Cisplatin	5.03363 ± 0.10899	(1.00)	8.48499 ± 0.19816	(1.00)
+ 0.4 μM ERK5-IN-1	4.90850 ± 0.07421	(1.03)	7.72168 ± 0.93519	(1.10)
	HEK293/Vector		HEK293/ABCB1	
Doxorubicin	0.12555 ± 0.00745	(1.00)	0.92581 ± 0.11863	(1.00)
+ 0.1 μM ERK5-IN-1	0.11298 ± 0.05714	(1.11)	0.29031 ± 0.06122 **	(3.19)
+ 0.2 μM ERK5-IN-1	0.10177 ± 0.00585 *	(1.23)	0.27352 ± 0.05309 **	(3.38)
+ 0.4 μM ERK5-IN-1	0.08744 ± 0.01173 **	(1.44)	0.19985 ± 0.00635 **	(4.63)
+ 10 μM Verapamil	0.13465 ± 0.02525	(0.93)	0.05056 ± 0.01142 **	(18.31)
Cisplatin	2.48910 ± 0.39760	(1.00)	3.58743 ± 0.43037	(1.00)
+ 0.4 μM ERK5-IN-1	2.58130 ± 0.27812	(0.96)	3.41328 ± 0.32569	(1.05)
	S1		S1-MI-80 (ABCG2)	
Mitoxantrone	0.04168 ± 0.00444	(1.00)	14.10528 ± 0.46080	(1.00)
+ 0.1 μM ERK5-IN-1	0.03865 ± 0.00137	(1.07)	12.15908 ± 2.02744	(1.16)
+ 0.2 μM ERK5-IN-1	0.03508 ± 0.00114	(1.19)	11.46149 ± 0.31168	(1.23)
+ 0.4 μM ERK5-IN-1	0.03265 ± 0.00026	(1.27)	11.45988 ± 1.95085	(1.23)
+ 2.5 μM FTC	0.03616 ± 0.00510	(1.15)	0.80305 ± 0.07874**	(17.56)

Cell viability was determined by MTT assay as described in Materials and Methods. Data represent means ± SD of at least three independent experiments. The fold-reversal of MDR was calculated by dividing the IC_50_ for cells with the chemotherapeutic agents in the absence of ERK5-IN-1 by that obtained in the presence of ERK5-IN-1. VRP (definite inhibitor of ABCB1) and FTC (definite inhibitor of ABCG2) were used as the positive control. * *P* < 0.05, ** *P* < 0.01, both for values versus that obtained in the absence of inhibitor

### ERK5-IN-1 reversed ABCB1-mediated MDR in vivo

**Fig. 2:**
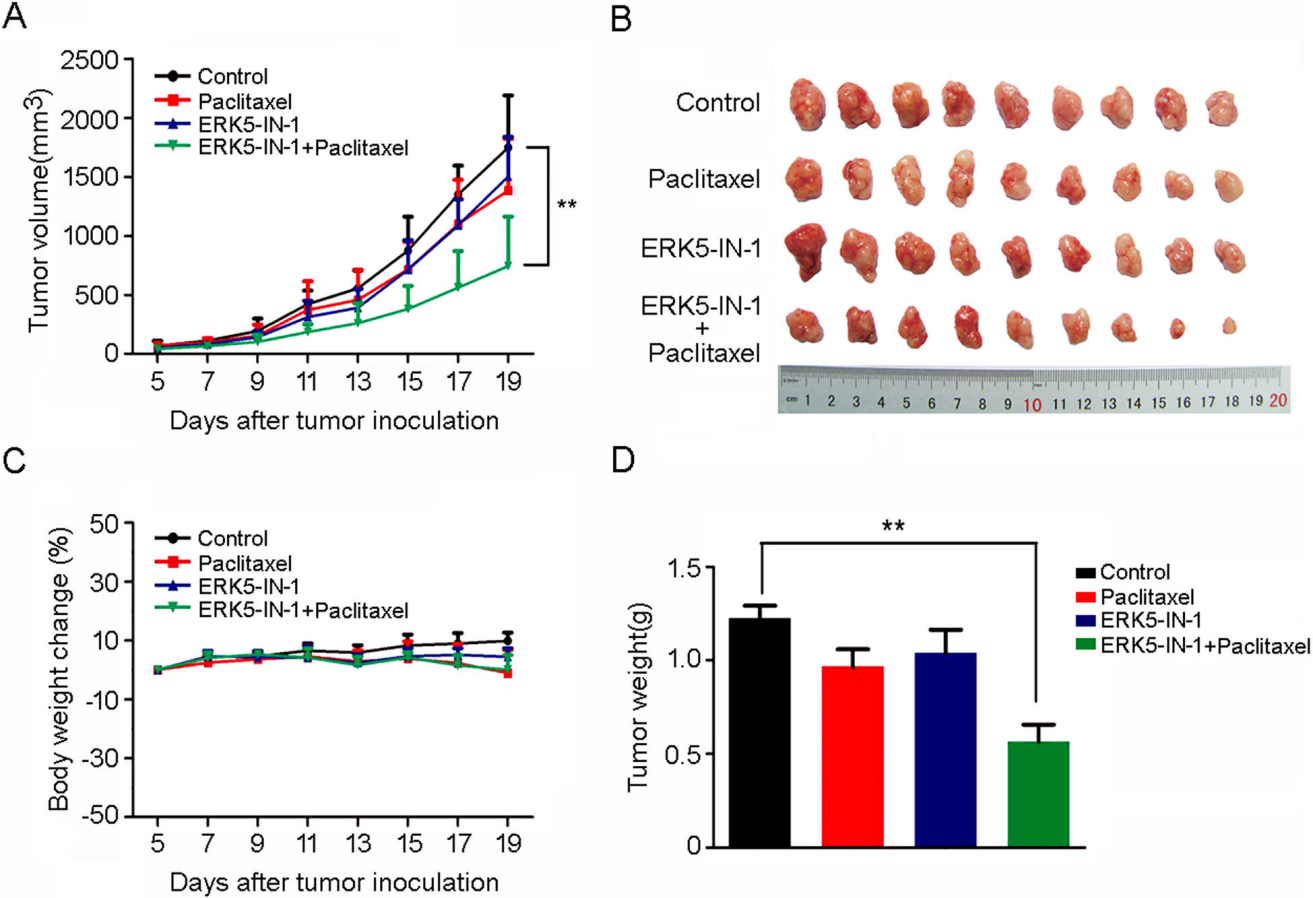
Potentiation of the antitumor effects of paclitaxel by ERK5-IN-1 in the KBv200 cell xenograft model. **a**, changes in tumor volume with time after cell inoculation. Data shown are means ± SD for each group of nine mice; **b**, tumor size, the photograph of xenografts was taken on the 19th day after implantation; **c**, changes of body weight after cell inoculation. Data shown are means ± SD for each group of nine mice after implantation; **d**, the average tumor weight of each group was calculated after the tumors were resected from mice. Data shown are mean ± SD for each group. Various treatments were given as follows: saline (q3d); paclitaxel (20 mg/kg, i.p. q3d); ERK5-IN-1 (25 mg/kg, gavage, q3d); ERK5-IN-1 (25 mg/kg, gavage, q3d, given 1 h before paclitaxel administration) and paclitaxel (20 mg/kg, i.p. q3d). **P < 0.01, significantly different from the control group

An established KBv200 cell xenograft model was used to evaluate the MDR reversal activity of ERK5-IN-1 in vivo. Paclitaxel and ERK5-IN-1 alone showed minimal inhibitory activity toward KBv200 tumors. However, the antitumor activity of paclitaxel was significantly enhanced when co-administered with ERK5-IN-1 and the inhibition rate was 46% (Fig. 2a, b and d). No significant body weight loss or mortality was observed, indicating that the combination regimen was tolerated by the mice (Fig. 2c).

### ERK5-IN-1 inhibited the transport function of ABCB1

**Fig. 3:**
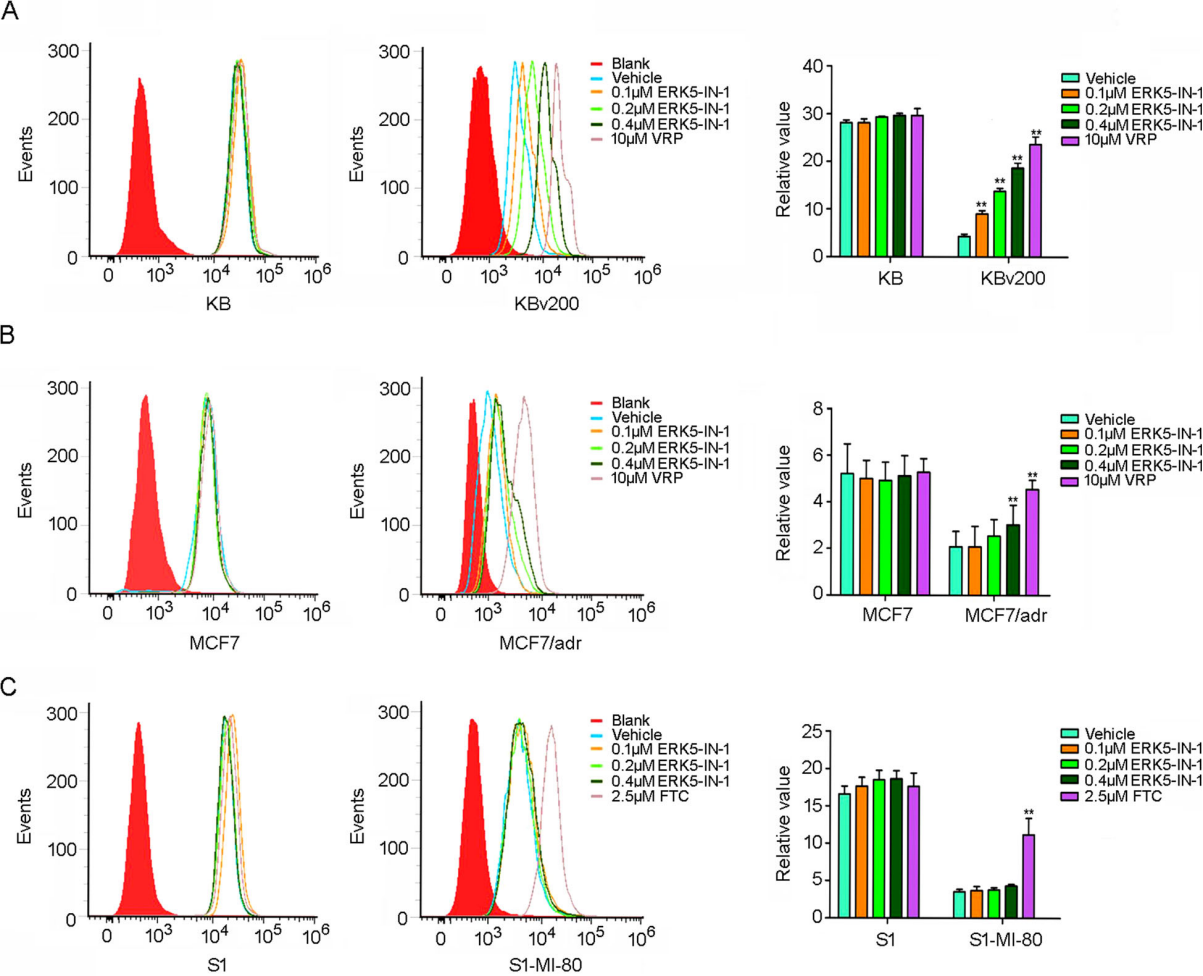
Effect of ERK5-IN-1 on the intracellular accumulation of Dox. The intracellular accumulations of Dox in: **a,** KB and KBv200 cells; **b**, MCF7 and MCF7/adr cells; **c**, S1 and S1-MI-80 cells were measured by flow cytometric analysis, respectively. Right column presents the quantification of Dox fluorescence, which was calculated by dividing the fluorescence intensity of each sample with that of MDR cells. Data shown are means ± SD of triplicate determinations. **P < 0.01 significantly different from control group

The results above indicated that ERK5-IN-1 effectively sensitized ABCB1 overexpressing MDR cells to chemotherapeutic agents. We further investigated the impact of ERK5-IN-1 on the transport function of ABCB1. The intracellular levels of Dox in MDR cells were much lower than the parental cells, whereas ERK5-IN-1 significantly increased the intracellular accumulation of Dox in a dose-dependent manner (Fig. 3a and b). However, no significant change in the intracellular accumulation of Dox was observed in S1-MI-80 cells suggesting that the reversal effects were dependent on the inhibition of ABCB1. By contrast, in the presence of FTC, cellular accumulation of Dox was significantly increased in S1-MI-80 cells (Fig. 3c).

### ERK5-IN-1 inhibited the efflux of Dox and stimulated the ATPase activity of ABCB1

**Fig. 4:**
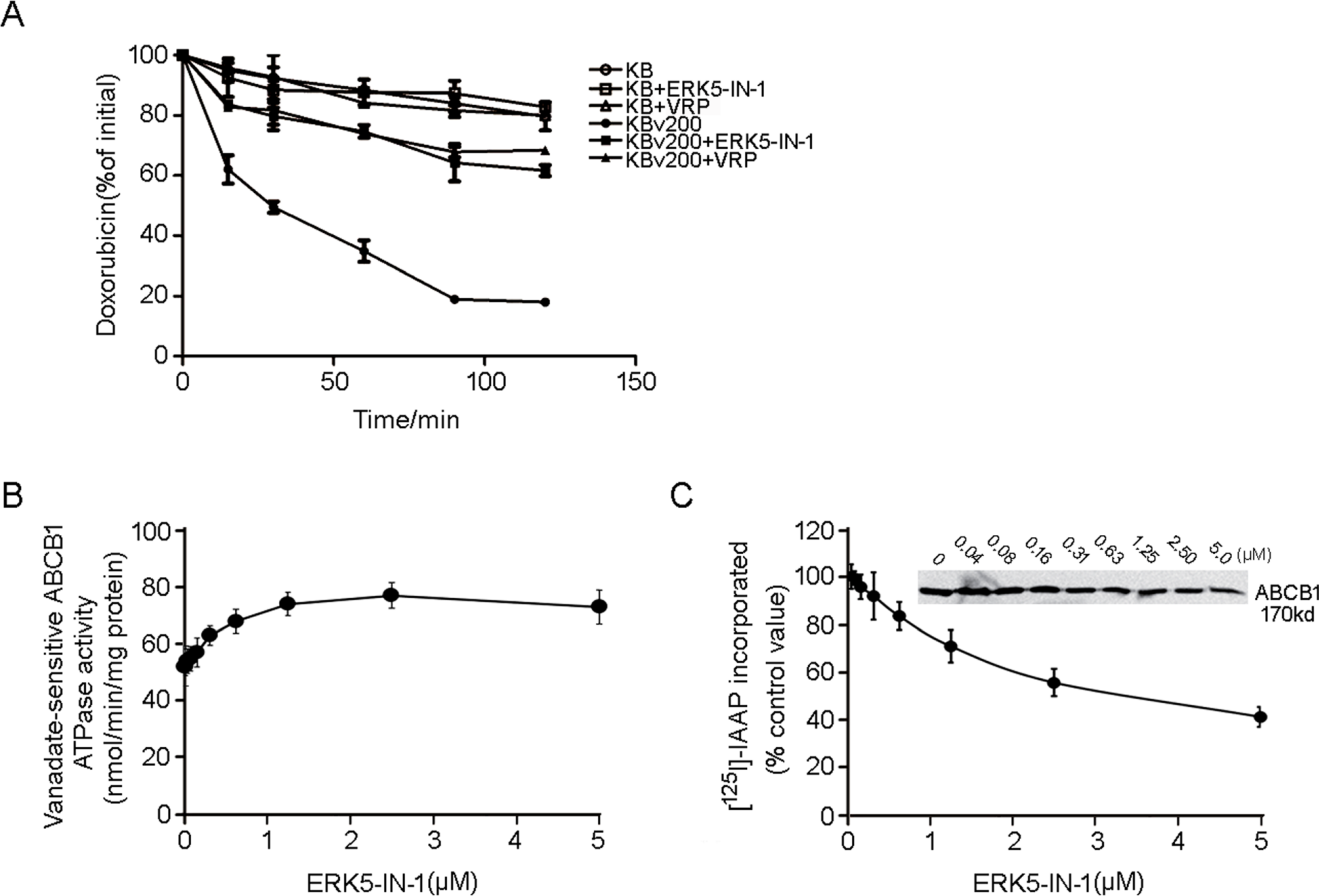
Effect of ERK5-IN-1 on efflux of Dox, ATPase activity and the [^125^I]-IAAP photolabeling of ABCB1. **a**, Time course of Dox efflux was measured in KB and KBv200 cells, with or without 0.4 μM ERK5-IN-1 or 10 μM VRP; **b**, the vanadate-sensitive ABCB1 ATPase activity in the presence of the indicated concentrations of ERK5-IN-1 was evaluated; **c**, ERK5-IN-1 competed for photolabeling of ABCB1 by [^125^I]-IAAP. The relative amount of [^125^I]-IAAP incorporated is plotted against the concentration of ERK5-IN-1 present. Each point represented the means ± SD of three independent experiments

Dox efflux experiment was carried out to testify if the increased accumulation of Dox was due to inhibition of efflux. As shown in Fig. 4a, the time course of intracellular Dox markedly decreased when KBv200 cells were incubated with 0.4 μM ERK5-IN-1, while no such effect was observed in KB cells. It is noteworthy that the inhibitory effect achieved by ERK5-IN-1 at 0.4 μM was comparable to that by VRP at 10 μM.

Given that the drug efflux function of ABCB1 is associated with ATP hydrolysis, we then measured the ATP hydrolysis activity mediated by ABCB1 when treated with different concentrations of ERK5-IN-1. As shown in Fig. 4b, ERK5-IN-1 stimulated the ATPase activity of ABCB1 in a dose-dependent manner. Evidence that ERK5-IN-1 interacts directly with ABCB1 was obtained by examining its effects on the inhibition of photoaffinity labeling of [^125^I]-IAAP. Crude membranes from High Five insect cells expressing ABCB1 were incubated with [^125^I]-IAAP and increasing concentrations (0–5 μM) of ERK5-IN-1. Our results showed that ERK5-IN-1 inhibited [^125^I]-IAAP binding to ABCB1 dose dependently. The 50% inhibitory concentration of [^125^I]-IAAP photoaffinity labeling of ABCB1 by ERK5-IN-1 was 3.7 μM (Fig. 4c). Thus, the [^125^I]-IAAP photolabeling of ABCB1 was slightly affected by ERK5-IN-1 at 0.4 μM, suggesting that either ERK5-IN-1 binds to a separate site, or that it has low-affinity for the IAAP binding site.

### ERK5-IN-1 did not affect ABCB1 expression

**Fig. 5:**
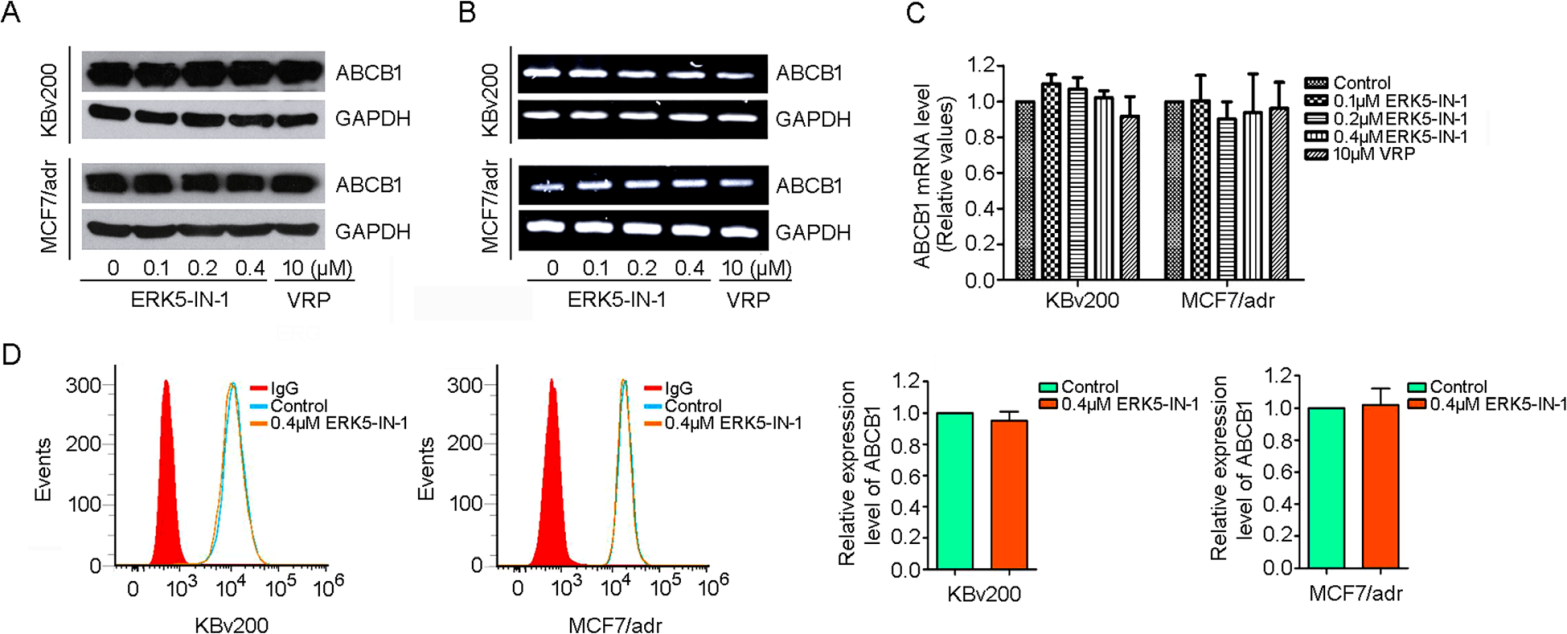
Effect of ERK5-IN-1 on the expression of ABCB1 in MDR cells. **a**, The protein and **b**, mRNA levels of ABCB1 were detected by Western blot and RT-PCR assays respectively; **c**, Real-time PCR assay was performed to quantify the mRNA levels of ABCB1 in KBv200 and MCF7/adr cells; **d**, the cell surface expression of ABCB1 in MDR cells was measured by flow cytometry. Columns, means of triplicate determinations; bars, SD

Considering that the reversal of MDR in cancers sometimes can be due to lowered expression of the protein, we further investigated the effect of ERK5-IN-1 on the expression of ABCB1 at protein and mRNA levels. Western blot and PCR analysis of KBv200 and MCF7/adr cells were performed after incubation for 48 h with ERK5-IN-1 at different concentrations and no decrease in ABCB1 expression was observed (Fig. 5a-c).

Cell surface expression and subcellular localization of ABCB1 in KBv200 and MCF7/adr cells were verified by flow cytometry and immunofluorescence. Our results showed that ERK5-IN-1 at 0.4 μM did not affect the cell surface expression of ABCB1 (Fig. 5d). The glycoprotein was predominantly localized in the transmembrane region in KBv200 cells and ERK5-IN-1 treatment did not change the distribution of ABCB1 (Additional file 1: Figure S1). These data indicate that ERK5-IN-1 exerts its MDR reversal activity via direct inhibition of ABCB1-mediated efflux, rather than modulation of its expression.

### ERK5-IN-1 did not block the phosphorylation of Akt and Erk1/2

**Fig. 6:**
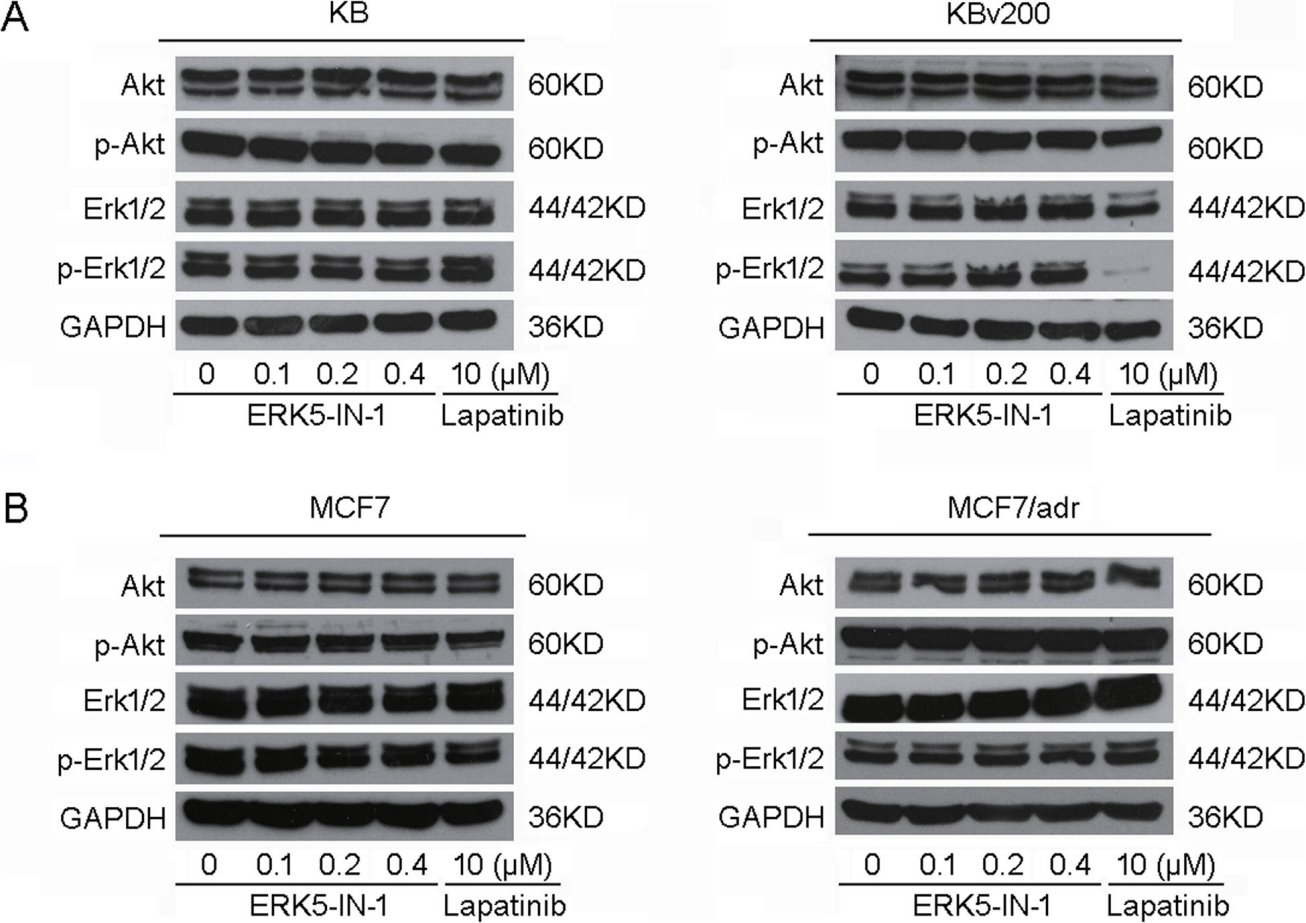
Effect of ERK5-IN-1 on the phosphorylation of Akt and Erk1/2. **a**, KB and KBv200, **b**, MCF7 and MCF7/adr cells were treated with ERK5-IN-1 at indicated concentrations for 24h. Lapatinib at 10 μM was used as positive control for the blockage of Akt and Erk1/2 phosphorylation. Representative results from three independent experiments were shown in each panel

Studies have shown that inhibition of Akt and Erk1/2, the downstream target kinases of ERK5-IN-1, may decrease resistance to antineoplastic drugs. To determine whether the MDR reversal activity of ERK5-IN-1 was related to inhibition of Akt or Erk1/2 phosphorylation, total and phosphorylated forms of Akt and Erk1/2 were measured after pretreatment with ERK5-IN-1 at concentrations used in the MTT assays. As shown in Fig. 6, ERK5-IN-1 treatment did not alter the total or phosphorylated forms of Akt and Erk1/2 in KB, KBv200, MCF7 and MCF7/adr cells. The results indicate that the MDR reversal activity of ERK5-IN-1 was independent of the blockage of Akt and Erk signaling pathways.

## Discussion

Constitutive and/or acquired MDR is a major obstacle to successful chemotherapy and overexpression of some of the ABC transporters was correlated with poor prognosis of patients [40]. Inhibition of ABC transporters appears a logical approach to circumvent MDR and improve patient’s outcome [41, 42]. To date, three generations of ABCB1 inhibitors have been tested in clinical trials. Both, the first-generation modulators (e.g., verapamil, quinidine, and cyclosporin A), and second generation agents (e.g., dexverapamil, valspodar and cinchonine) in combination with chemotherapy failed to improve outcomes in clinical trials due to unacceptable toxicities and unexpected pharmacokinetic interactions, respectively. Consequently, the third-generation agents (e.g., laniquidar, zosuquidar and biricodar) were developed using structure-activity, which showed high affinity and low pharmacokinetic interaction due to a limited CYP3A inhibition. Nevertheless disappointingly, no overall satisfactory results have been obtained in clinical trials. Studies in the development of fourth-generation agents including natural compounds, peptidomimetics and surfactants are still ongoing. The development of safe and effective MDR-reversing agents still remains an unmet need in clinic.

Tyrosine kinase inhibitors (TKIs) such as imatinib, gefitinib and erlotinib are small molecules developed to inhibit the uncontrolled activity of various tyrosine kinases involved in cancer [43, 44]. Many of them are either substrates, inhibitors or both for ABC transporters [22, 45, 46]. Some combination therapies of TKIs with cytotoxic drugs have been performed to overcome MDR in the clinic, including the combination of gefitinib with 5-fluorouracil, leucovorin, and irinotecan in patients with colorectal cancer, the combination of imatinib and cytarabine in newly diagnosed patients with chronic myeloid leukemia, and the combination of gemcitabine/cisplatin with gefitinib in NSCLC [47–49].

ERK5, also known as BMK1, is the least studied member of mitogen-activated protein kinases (MAPKs) family, but has been reported to play an important role in tumor neovascularization, tumor cell invasion and migration. Scientists have also identified MEK5/ERK5 pathway as a novel target implicated in chemoresistance by gene expression profiling analysis [50]. ERK5-IN-1 was discovered as a novel ERK5 inhibitor scaffold, which shows excellent kinase selectivity and extraordinary anticancer activities. In this study, we explored the effects of ERK5-IN-1 on reversal of MDR and its interaction with ABC transporters.

The in vitro modulating potency was first studied by the potentiation of cytotoxic drug activity using the MTT assay. The cytotoxic activity of ERK5-IN-1 were evaluated in vitro using a panel of cancer cell lines stabling expressing ABCB1 or ABCG2 and ERK5-IN-1 at non-toxic concentrations up to 0.4 μM were used for reversal assay in vitro (Fig. 1). Our results showed that significant reversal of resistance in ABCB1-overexpressing cells to chemotherapy drugs was achieved in the presence of ERK5-IN-1, but no such effect in the drug-sensitive parental cell lines was observed. Additionally, ERK5-IN-1 did not affect the cytotoxicity of non-Pgp substrates such as cisplatin (Table 1). However, parallel results were not observed in S1-MI-80, HL60/adr, HEK293/MRP7–2 and SW1573/2R120 cells indicating that ERK5-IN-1 could not reverse ABCG2-, ABCC1-, MRP7- and LRP-mediated MDR (Table 1 and S1).

The promising activity of ERK5-IN-1 demonstrated in vitro was further confirmed in vivo, we examined the effect of ERK5-IN-1 on enhancing the antitumor activity of paclitaxel in KBv200 xenograft model. Consistent with the in vitro results, our results indicated that ERK5-IN-1 in combination with paclitaxel resulted in markedly enhanced antitumor activity of paclitaxel in mice (Fig. 2).

To elucidate the mechanism of reversal of ABCB1-mediated MDR by ERK5-IN-1, ABCB1 transport activity and expression were examined. Consistent with the high expression level of ABCB1, KBv200 and MCF7/adr cells showed lower intracellular accumulation of Dox than KB and MCF7 cells. ERK5-IN-1 treatment effectively inhibited the efflux function of ABCB1 and significantly increased the intracellular accumulation of Dox, while no significant change was found in the parental KB, MCF7, S1 and ABCG2-overexpressing S1-MI-80 cells (Fig. 3).

ABC transporters exert their drug efflux function by utilizing energy derived from the hydrolysis of ATP, and thus the change of ATPase activity of the transporters directly reflects their transport activity [51]. We found that ERK5-IN-1 significantly stimulated ATPase activity at low concentrations (0-1 μM). This is further confirmed by the results that ERK5-IN-1 competed with [^125^I]-IAAP for the photoaffinity labeling of ABCB1 (Fig. 4). These data suggested that ERK5-IN-1 did circumvent MDR by inhibiting the efflux function of ABCB1. However, it’s worth noting that that ERK5-IN-1 was less potent in inhibiting photolabeling of ABCB1 with [^125^I]-IAAP than other TKIs (e.g., lapatinib and neratinib) which could also reverse ABCB1-mediated MDR effectively [23, 52]. This indicates that either ERK5-IN-1 binds to a separate site, or that it has low-affinity for the IAAP binding site. In drug efflux assay and uptake assay ERK5-IN-1 at 0.4 μM showed comparable results to verapamil, while in cytotoxicity assay verapamil showed much higher enhancement than ERK5-IN-1 at 0.4 μM. The divergence between these two results might be partly explained by the complex competition behavior exhibited by ERK5-IN-1.

The reversal of MDR may be achieved by downregulation of the ABC transporter expression, thus the possible regulation of expression of ABCB1 by ERK5-IN-1 was also examined [53, 54]. Our results showed that neither the expression of ABCB1 at protein or mRNA level nor the cell surface localization of ABCB1 was affected in the presence of ERK5-IN-1 (Fig. 5 and Additional file 1: Figure S1).

**Fig. 7:**
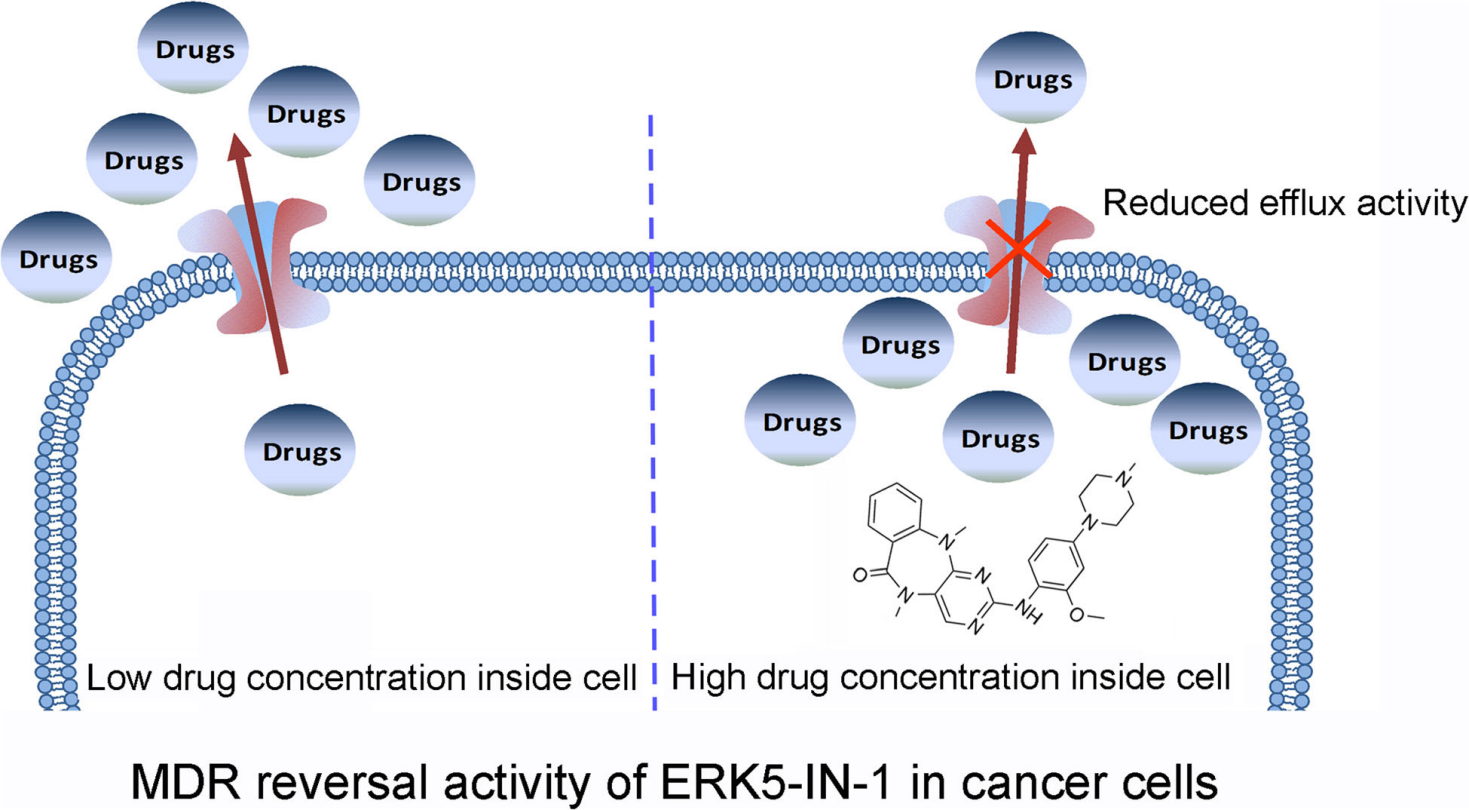
Schematic diagram of ERK5-IN-1 reversing MDR. Left, In the absence of ERK5-IN-1, ABCB1 transporters utilize energy derived from the hydrolysis of ATP to efflux their substrates crossing the membrane; Right, In the presence of ERK5-IN-1, ERK5-IN-1 increases the intracellular accumulation of the substrate drugs by blocking the efflux function of ABCB1

As a kinase inhibitor, ERK5-IN-1 inhibited cell proliferation via blocking downstream signaling pathways such as phosphorylation of Akt and Erk1/2, which could also enhance the efficacy of chemotherapeutic agents. To elucidate whether the reversal effect in ABCB1-mediated MDR cells was related to the altered activation of downstream targets, cells were pretreated with ERK5-IN-1 and then subjected to Western blot analysis. Our data revealed no significant alteration of the phosphorylation of Akt and Erk1/2 in the tested cell lines, suggesting that the reversal activity of ERK5-IN-1 was not associated with the inhibition Akt and Erk pathways (Fig. 6). A schematic model illustrating the reversal of MDR by ERK5-IN-1 is shown in Fig. 7.

## Conclusions

Reversal of MDR by ERK5-IN-1 was through selective and potent inhibition of ABCB1 function. These findings provide strong support for further development of ERK5-IN-1 or its derivates as candidate inhibitors of MDR in cancer treatment.

## Supplementary information

**Additional file 1: Figure S1.** Effect of ERK5-IN-1 on the subcellular distribution of ABCB1. KBv200 cells were treated with or without ERK5-IN-1 at 0.4 μM for 48 h. The subcellular localization pattern of ABCB1 was evaluated using confocal laser scanning microscopy. ABCB1 (red) and nuclei (DAPI, blue) were visualized

**Additional file 2: Table S1.** Effect of ERK5-IN-1 on reversing ABCC1, MRP7 and LRP-mediated drug resistance

## Data Availability

The datasets used and/or analysed during the current study are available from the corresponding author on reasonable request. The key data have been uploaded to the Research Data Deposit Platform public database (www.researchdata.org.cn), No. RDDB2019000743.

## Abbreviations

ABC: Adenosine triphosphate (ATP)-binding cassette
ABCB1: ATP-binding cassette subfamily B member 1
ABCC1: Human Multidrug resistance-associated protein 1
ABCG2: ABC transporter subfamily G member 2
DMSO: Dimethyl sulfoxide
Dox: Doxorubicin
ERK5: Extracelluar signal regulated kinase 5
FTC: Fumitremorgin C
IAAP: Iodoarylazidoprazosin
LRP: Lung resistance-related protein
MDR: Multidrug resistance
MRP7: Multidrug resistance protein 7
MTT: 3-(4,5-dimethylthiazol-2yl)-2,5-diphenyltetrazoliumbromide
PCR: Polymerase chain reaction
