# Supplementary figures and images for: Reversal of ABCB1-related multidrug resistance by ERK5-IN-1
Source: J Exp Clin Cancer Res. 2020 Mar 12;39:50. doi: 10.1186/s13046-020-1537-9 (PMC7066765; doi:10.1186/s13046-020-1537-9)

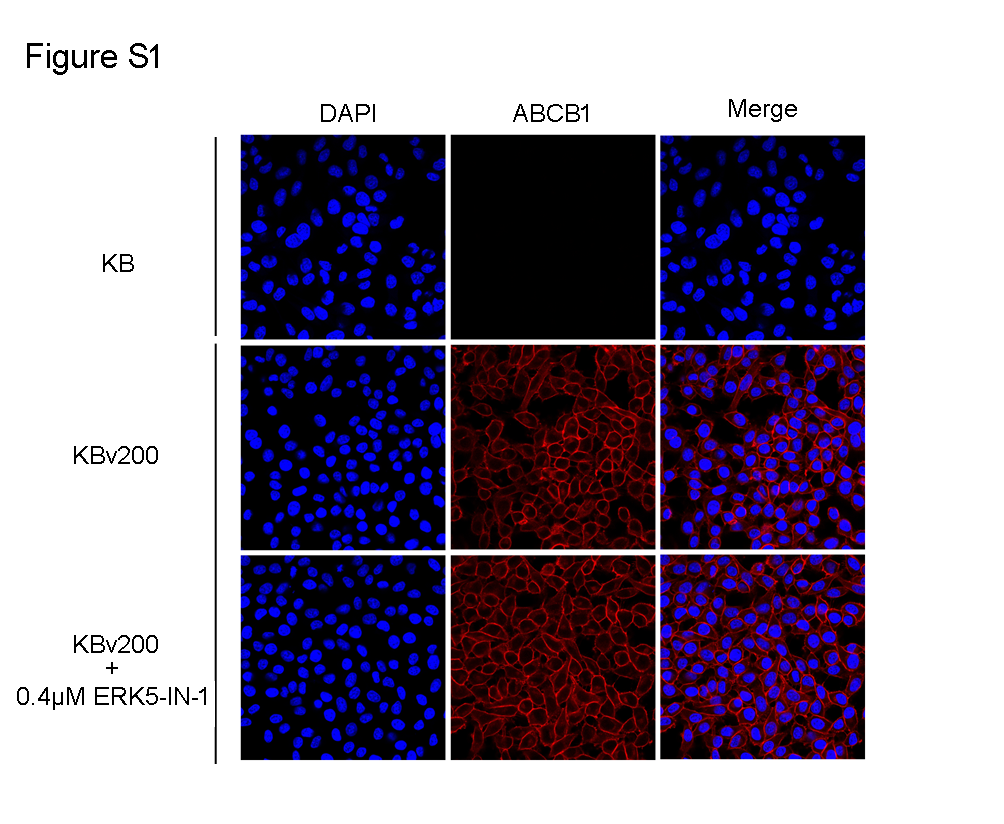

Supplement: Supplementary file 1 — Additional file 1: Figure S1. Effect of ERK5-IN-1 on the subcellular distribution of ABCB1. KBv200 cells were treated with or without ERK5-IN-1 at 0.4 μM for 48 h. The subcellular localization pattern of ABCB1 was evaluated using confocal laser scanning microscopy. ABCB1 (red) and nuclei (DAPI, blue) were visualized [file 13046_2020_1537_MOESM1_ESM.tif]
