# Supplementary material for: Reversal of ABCB1-related multidrug resistance by ERK5-IN-1
Source: J Exp Clin Cancer Res. 2020 Mar 12;39:50. doi: 10.1186/s13046-020-1537-9 (PMC7066765; doi:10.1186/s13046-020-1537-9)
Supplement: Supplementary file 2 — Additional file 2: Table S1. Effect of ERK5-IN-1 on reversing ABCC1, MRP7 and LRP-mediated drug resistance [file 13046_2020_1537_MOESM2_ESM.docx]

**Table S1. Effect of ERK5-IN-1 on reversing ABCC1, MRP7 and LRP-mediated drug resistance**

| Compounds | IC_50_ ± SD (μmol/L) (fold-reversal) | | | | |
| --- | --- | --- | --- | --- | --- |
|  | HL60 | | HL60/adr(ABCC1) | | |
| Doxorubicin | 0.0120 ± 0.0027 | (1.00) | 2.6740 ± 0.8125 | | (1.00) |
| + 0.1 μM ERK5-IN-1 | 0.0139 ±0.0050 | (0.87) | 2.6062 ± 0.3715 | | (1.03) |
| + 0.2 μM ERK5-IN-1 | 0.0132 ± 0.0034 | (0.91) | 1.7925 ± 0.2474 | | (1.49) |
| + 0.4 μM ERK5-IN-1 | 0.0145 ± 0.0013 | (0.83) | 2.1013 ± 0.3290 | | (1.27) |
| + 50 μM MK571 | 0.0147 ± 0.0016 | (0.82) | 0.4027 ± 0.0732** | | (6.64) |
|  | HEK293/Vector | | HEK293/MRP7-2 | | |
| Paclitaxel | 0.0117 ± 0.0014 | (1.00) | 0.2576 ± 0.0517 | | (1.00) |
| + 0.1 μM ERK5-IN-1 | 0.0166 ± 0.0028 | (0.70) | 0.2966 ± 0.0492 | | (0.87) |
| + 0.2 μM ERK5-IN-1 | 0.0168 ± 0.0019 | (0.70) | 0.2378 ± 0.0163 | | (1.08) |
| + 0.4 μM ERK5-IN-1 | 0.0150 ± 0.0015 | (0.78) | 0.2698 ± 0.0392 | | (0.95) |
|  | SW1573 | | SW1573/2R120(LRP) | | |
| Doxorubicin | 0.1132 ± 0.0151 | (1.00) | | 1.4091 ± 0.1467 | (1.00) |
| + 0.1 μM ERK5-IN-1 | 0.1209 ± 0.0069 | (0.94) | | 1.3594 ± 0.0264 | (1.04) |
| + 0.2 μM ERK5-IN-1 | 0.1217 ± 0.0025 | (0.93) | | 1.4521 ± 0.4034 | (0.97) |
| + 0.4 μM ERK5-IN-1 | 0.1504 ± 0.0233 | (0.75) | | 1.1606 ± 0.2926 | (1.21) |

Cell viability was determined by MTT assay as described in Materials and Methods. Data represent means ± SD of at least three independent experiments. The fold-reversal of MDR was calculated by dividing the IC_50_ for cells with the chemotherapeutic agents in the absence of ERK5-IN-1 by that obtained in the presence of ERK5-IN-1. MK571 (definite inhibitor of ABCC1) was used as the positive control. ** *P* < 0.01, both for values versus that obtained in the absence of inhibitor.
